# Host plant use of a polyphagous mirid, *Apolygus lucorum*: Molecular evidence from migratory individuals

**DOI:** 10.1002/ece3.5660

**Published:** 2019-09-21

**Authors:** Qian Wang, Weifang Bao, Qian Zhang, Xiaowei Fu, Yizhong Yang, Yanhui Lu

**Affiliations:** ^1^ College of Horticulture and Plant Protection Yangzhou University Yangzhou China; ^2^ State Key Laboratory for Biology of Plant Diseases and Insect Pests Institute of Plant Protection Chinese Academy of Agricultural Sciences Beijing China; ^3^ College of Agriculture and Food Science Zhejiang A & F University Hangzhou China

**Keywords:** DNA barcoding, feeding ecology, gut content, insect–plant interaction, Miridae

## Abstract

While the host plant use of insect herbivores is important for understanding their interactions and coevolution, field evidence of these preferences is limited for generalist species. Molecular diet analysis provides an effective option for gaining such information, but data from field‐sampled individuals are often greatly affected by the local composition of their host plants. The polyphagous mirid bug *Apolygus lucorum* (Meyer‐Dür) seasonally migrates across the Bohai Sea, and molecular analysis of migrant bugs collected on crop‐free islands can be used to estimate the host plant use of *A. lucorum* across the large area (northern China) from where these individuals come. In this study, the host plant use of *A. lucorum* adults was determined by identifying plant DNA using a three‐locus DNA barcode (*rbc*L, *trn*H‐*psb*A, and ITS) in the gut of migrant individuals collected on Beihuang Island. We successfully identified the host plant families of *A. lucorum* adults, and the results indicated that captured bugs fed on at least 17 plant families. In addition, gut analyses revealed that 35.9% of *A. lucorum* individuals fed on multiple host plants but that most individuals (64.1%) fed on only one plant species. Cotton, *Gossypium hirsutum* L., DNA was found in 35.8% of the *A. lucorum* bugs examined, which was much higher than the percentage of bugs in which other host plants were found. Our work provides a new understanding of multiple host plant use by *A. lucorum* under natural conditions, and these findings are available for developing effective management strategies against this polyphagous pest species.

## Graphical abstract

We determined the host plant use of its adults by identifying plant DNA in the gut of migrant individuals collected on the crop‐free island. The results indicated that captured bugs fed on at least 17 plant families, 35.9% of bugs fed on multiple host plants, and cotton plant DNA was found in 35.8% of the bugs examined. It provides a new understanding of host plant use by *A. lucorum* across the large area (northern China) from where these migrant individuals come.

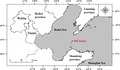

## 1 INTRODUCTION

The interaction between insect herbivores and plants greatly drives their coevolution (Becerra, 2003; Berenbaum, 2001; Gaunt & Miles, 2002; Hare, 2012; Schuman & Baldwin, 2015; Wu & Baldwin, 2010). Accurately determining the complex associations between insect herbivores and host plants is crucial to understanding how such ecological interactions are established (García‐Robledo, Erickson, Staines, Erwin, & Kress, 2013). Numerous studies have examined the diet of specialist herbivores to detect specific behavioral and physiological adaptations between herbivore species and their host plants (Johnson & Nicolson, 2001; Moore et al., 1987; Schlein & Muller, 1995; Zhang et al., 2019). Generalist herbivores have a wide range of host plant species and rarely show specific adaptations to particular plants (Barros, Torres, Ruberson, & Oliveira, 2010; Franzke, Unsicker, Specht, Köhler, & Weisser, 2010; Hereward & Walter, 2012; Joern, 1979). However, not all the plant species found in the habitats of generalist herbivores can be utilized, and the diets of these herbivores, while diversified, are still somewhat selective (Ibanez et al., 2013). Direct observations of herbivory in the field are problematic in habitats that are difficult to access, such as the forest canopy or underground, and are also greatly limited by the ability of the researcher to correctly identify the species involved in the interactions. Since the observation of feeding behavior cannot produce a clear picture of a generalist herbivore's entire host plant range, a more accurate method for determining the feeding history and alternative (noncrop) host plants of generalist herbivores is needed.

DNA barcoding uses short DNA sequence markers for the taxonomic identification of species (Hebert, Penton, Burns, Janzen, & Hallwachs, 2004; Heise, Babik, Kubisz, & Kajtoch, 2015), which can overcome the problems associated with more conventional methodologies, as it can enable rapid, sensitive, and accurate plant species identification by detecting host plant‐specific DNA extracted from herbivorous insects (Traugott, Kamenova, Ruess, Seeber, & Plantegenest, 2013; Valentini, Pompanon, & Taberlet, 2009). For these reasons, this technique has attracted increasing attention in the past several years as a method for determining the dietary composition of herbivores (Erickson et al., 2017; García‐Robledo et al., 2013; Heise et al., 2015; Jurado‐Rivera, Vogler, Reid, Petitpierre, & Gomez‐Zurita, 2009; Navarro, Jurado‐Rivera, Gómez‐Zurita, Lyal, & Vogler, 2010; Staudacher, Wallinger, Schallhart, & Traugott, 2011). In these studies, specific plant barcode regions (e.g., *rbc*L and *trn*L) were amplified and compared with known DNA sequences in GenBank using BLAST (Altschul, Gish, Miller, Myers, & Lipman, 1990), which could allow for the identification of unknown ingested host plant species (Jurado‐Rivera et al., 2009; Navarro et al., 2010). Molecular markers have shown great potential for identifying the diets of insect herbivores at the taxonomic levels of family and genus (Jurado‐Rivera et al., 2009; Navarro et al., 2010) and even at the species level (García‐Robledo et al., 2013). In species‐level identification, a comprehensive DNA sequence database of the target community is required, and improved DNA extraction techniques and multiple molecular markers will help increase the efficiency of species discrimination. For example, García‐Robledo et al. (2013) accurately identified the dietary breadth of leaf‐rolling beetles in a tropical rain forest in Costa Rica by three DNA barcode loci (i.e., *rbc*L, ITS2, and *trn*H‐*psb*A). Hereward and Walter (2012) used a *trn*L‐*trn*F fragment to identify the plant species fed on by the green mirid *Creontiades dilutus* in northeastern Australia and found that the mirid individuals frequently fed on more plants than the species from which they were collected. This DNA‐based technique allows us to better understand the feeding activities of insect herbivores instead of needing to make direct feeding observations (Kiston et al., 2013; La Cadena, Papadopoulou, Maes, & Gómez‐zurita, 2015; Wang, Bao, Zeng, Yang, & Lu, 2016). Moreover, as DNA barcoding techniques are less targeted, they can reduce the risk of overlooking the trophic relationships of generalist herbivores (Kishimoto‐Yamada et al., 2013). Many unexpected trophic associations have been discovered with the application of molecular methods (Jurado‐Rivera et al., 2009; La Cadena et al., 2015). Jurado‐Rivera et al. (2009) sequenced the *trn*L gene in the plant DNA extracted from 78 Chrysomelinae samples and found that Chrysomelinae fed on 13 plant families, with a preference for Australian radiations of Myrtaceae and Fabaceae; moreover, 40% of the host plants were previously undocumented, including rare or nondominant plants that are often missed or ignored. Unexpected trophic interaction may be particularly common in polyphagous organisms, especially those that are studied primarily as crop pests, where alternative hosts may be largely ignored by researchers.

The polyphagous mirid bug *Apolygus lucorum* (Meyer‐Dür) (Hemiptera: Miridae) with more than 200 species of recorded host plants is the dominant pest mirid of cotton (*Gossypium hirsutum* L.), fruit trees, and many other crops in China (Lu, 2008; Lu, Wu, Jiang, et al., 2010). *A. lucorum* nymphs and adults feed on multiple vegetative and reproductive tissues of their host plants via piercing and sucking mouthparts (Jiang, Lu, & Zeng, 2015; Zhang, Lu, & Liang, 2013). They use stylets to lacerate the plant cells while secreting a watery saliva (including a high diversity of digestive enzymes) into the ruptured cell and then ingest the resultant lacerated/macerated “soup” (Backus, Cline, Ellerseick, & Serrano, 2007). This feeding strategy usually leads to the necrosis and discoloration of plant tissue, the formation of bushy plants, the abscission of flower buds, and the distortion of mature fruits (Jiang et al., 2015; Shackel et al., 2005), which often greatly reduces yield and quality when the population of *A. lucorum* is large (Lu & Wu, 2008). Damage symptoms usually appear approximately one week after mirid bug feeding (Jiang et al., 2015; Zhang et al., 2013), and adults frequently move between different host plants (Pan, Lu, Wyckhuys, & Wu, 2013; Wang, Bao, Yang, Yang, & Lu, 2018). The relatively cryptic feeding habits and high mobility of this species make it difficult to precisely assess its host plant use with field population surveys. However, plant identification using plant DNA barcode loci and the well‐studied plant–herbivore system allows us to accurately identify insect diets (Kress & Erickson, 2007; Li et al., 2011).

In molecular dietary analysis of herbivorous insects, the information on host plant use obtained from field‐sampled individuals is likely to vary greatly among different sampling locations, which usually differ in host plant composition (Kishimoto‐Yamada et al., 2013; Wang et al., 2016). Hence, the design of the sampling program is vital and plays an important role in lessening the possible overrepresentation of particular locally abundant hosts in data from field‐collected insect individuals (e.g., Hereward, DeBarro, & Walter, 2013). For adult *A. lucorum*, 10‐day‐old mated females showed a maximum flight distance of 111.4 km during a 24‐hr period in flight mill assays, indicating that *A. lucorum* adults possess strong potential for long‐distance flight (Lu, Wu, & Guo, 2007). An 11‐year searchlight trapping and radar observation study on an isolated island (Beihuang) in the center of the Bohai Gulf found that *A. lucorum*, a migratory species, travels at least 40–60 km from land (Fu et al., 2014). As almost no crops are grown on Beihuang Island, it is an ideal site to collect migrating *A. lucorum* from northern China without a strong local influence of dietary breadth. Further analysis of these migrant adults collected from Beihuang Island might explain the host plant use of *A. lucorum* in northern China while eliminating the bias of specific sampling sites.

In this study, we first collected migrant *A. lucorum* adults using light traps on the island of Beihuang, sequenced short stretches of plant‐specific genes (i.e., *rbc*L, ITS, and *trn*H‐*psb*A) from the gut contents of each *A. lucorum* adult, and then compared the resultant DNA sequences with GenBank sequences to confirm the host plant species.

## 2 MATERIALS AND METHODS

### 2.1 Insect collection

**Figure 1:**
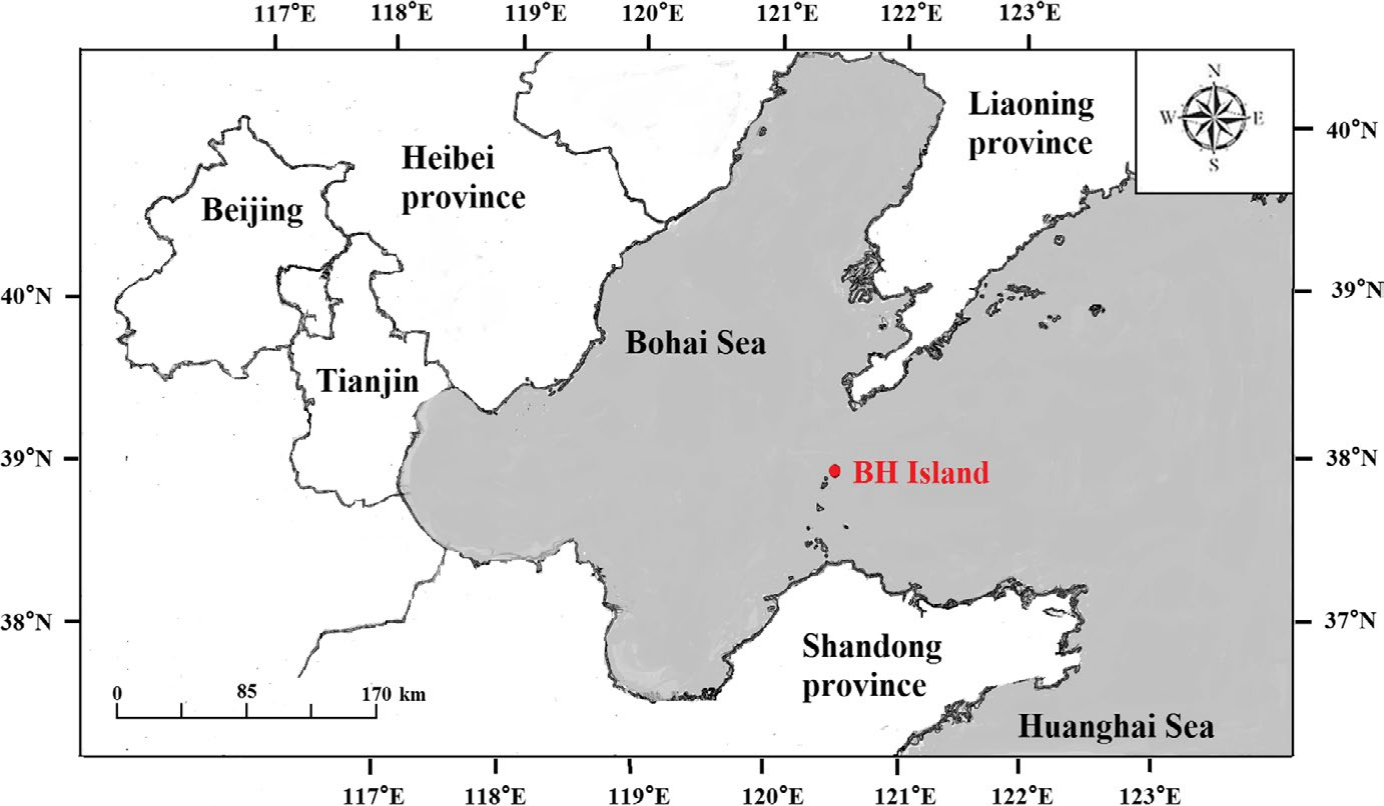
Site (Beihuang Island) for migrant adult sampling

*Apolygus lucorum* adults were collected on the island of Beihuang (BH, 38°240 N; 120°550 E; Figure 1) in the Bohai Strait. The island is located approximately 40–60 km from the land of northern China (Cheng, Feng, & Wu, 2005; Feng, Wu, Cheng, & Guo, 2004; Feng, Wu, Cheng, & Guo, 2007; Liu, Fu, Feng, Liu, & Wu, 2015). Collections were made using a light trap every night from June to August in 2012, 2014, and 2015. *Apolygus lucorum* adults were collected by a vertically pointed searchlight trap from sunset to sunrise, except during power outages or periods of heavy rain. The searchlight trap (model DK.Z.J1000B/t, 65.2 cm in diameter, 70.6 cm in height, and 30 cm in spread angle) was equipped with a 1,000‐W metal halide lamp (model JLZ1000BT; Shanghai Yaming Lighting Co., Ltd.) mounted on the top of a house (500‐m elevation). We removed trapped *A. lucorum* individuals from the nylon net (60 mesh) bags at 6:00 a.m., after which they were identified and transferred into a 1.5‐ml tube and stored in a freezer (at −20°C) for later extraction.

### 2.2 Insect DNA extraction

DNA was extracted from whole adult of *A. lucorum* following a previously described CTAB‐based protocol (Wallinger et al., 2013). Before DNA extraction, each adult was cleaned of plant material potentially adhering to its body surface following a modified method (Greenstone, Payton, Weber, & Simmons, 2014; Remén, Krüger, & Cassel‐Lundhagen, 2010; Wallinger et al., 2013). Specifically, we placed each *A. lucorum* in 1 ml of 1%–1.5% sodium hypochlorite (Beijing Chemical Works) for 5 s and then rinsed it twice with molecular analysis‐grade water (Wang, Bao, Wu, Yang, & Lu, 2017). To check for cross‐sample contamination, two extraction‐negative controls were included in each batch of 24 samples.

### 2.3 PCR assays

Three plant DNA barcode loci (i.e., *rbc*L, ITS, and *trn*H‐*psb*A) were sequenced for each sample to increase the recovery of intact sequences from potentially highly degraded plant DNA from insect gut contents (Kress & Erickson, 2007; Kress et al., 2009; Li et al., 2011). The nucleotide sequences (5′ to 3′) of the primers are listed in Table S1. PCR was performed in 25 μl of solution containing 4 μl of DNA solution (10 ng/μl), 0.75 μl of each primer (10 μM), 2.5 μl of 10 × Taq buffer (TransGen Biotech), 0.5 μl of dNTP (2.5 mM), 0.25 μl of Easy Taq (5 units/μl) (TransGen Biotech), 0.75 μl of each primer (10 μM), and 16.25 μl of autoclaved distilled water. The PCRs were performed in Veriti 96‐well thermal cyclers (Applied Biosystems). The thermocycling program was as follows: 95°C for 10 min, followed by 35 cycles of 95°C for 30 s, 56°C for 30 s, and 72°C for 1 min, and a final extension of 72°C for 10 min. Amplified products (20 μl) were analyzed by electrophoresis on a 2% agarose gel in TAE buffer (40 mmol/L Tris‐acetate, 2 mmol/L Na_2_EDTA·H_2_O) and visualized with a UV transilluminator. Two positive [mungbean (*Vigna radiata* (L.) Wilczek) plant DNA] and two negative controls (PCR‐grade water instead of extracted insect DNA) were included in each PCR assay to determine amplification success and DNA carryover contamination, respectively.

### 2.4 Cloning and DNA sequencing

PCR products were purified with a gel extraction kit (Tiangen) and ligated into pGEM‐T cloning vector (Promega). Successful insertion was verified by PCR with the M13 forward (5′‐GTTTTCCCAGTCACGAC‐3′) and M13 reverse primers (5′‐CAGGAAACAGCTATGAC‐3′), and Sanger sequencing was performed at Biomed (Beijing, China). A total of 30 clones were sequenced per sample.

### 2.5 Identification of *A. lucorum* diets using molecular markers

*Apolygus lucorum* gut content DNA sequence identifications were performed using BLAST against GenBank using the default search parameters (Altschul et al., 1990). Each unknown DNA sequence from the gut contents was identified to the species level only when it was nearly completely consistent with the best hit of the query sequences (percent identity > 99%). In cases where top BLAST scores were equal for species from different genera within the same genus, we identified such interactions to the genus level. Identification of DNA sequences at the family level was similar to the method used for genus identification. Sequences from gut contents that did not match any of the plant DNA sequences in the DNA barcode library were scored as unidentified.

### 2.6 Data analysis

Differences in the detected host plants of *A. lucorum* in different years and months were compared via two‐factor nonrepetitive variance analysis via the GLM (proc glm) process step in SAS 9.30 software (SAS Inc). Before the analysis, the detection rate data were subjected to inverse sine transformation to improve normality.

## 3 RESULTS

### 3.1 Inferred plant families

Two hundred and seventy‐eight high‐quality sequences were detected among the 156 *A. lucorum* individuals, including 29 *rbc*L sequences, 137 ITS sequences, and 112*trn*H‐*psb*A sequences, which were discriminated into 33 OTUs that were assigned to at least 17 families (Table 1). Among the *rbc*L sequences amplified from insects, the amplification success rate of plant DNA in *A. lucorum* was relatively low (15.4%), indicating that 93.1% and 6.9% of the sequences were successfully identified to the plant genus and species levels, respectively. The ITS and *trn*H‐*psb*A primers successfully amplified plant DNA in a higher percentage of *A. lucorum* individuals (ITS: 57.1%; *trn*H‐*psb*A: 42.1%) with species‐level identifications (ITS: 73.0%; *trn*H‐*psb*A: 40.2%) and genus‐level identifications (ITS: 23.4%; *trn*H‐*psb*A: 53.6%) (Table 2).

**Table 1:** The inferred host plants of *Apolygus lucorum* through use of three DNA barcodes

DNA barcodes	Number of sequences	Inferred plants	Inferred plant family
*rbc*L	1	*Amorpha fruticosa* L.	Leguminosae
	4	*Acacia*	Mimosaceae
	1	*Citrus*	Mimosaceae
	16	*Ulmus*	Ulmaceae
	6	*Ricinus*	Euphorbiaceae
	1	*Helianthus*	Asteraceae
ITS	86	*Gossypium hirsutum* L.	Malvaceae
	2	*Triticum*	Gramineae
	2	*Flueggea*	Euphorbiaceae
	1	*Vigna unguiculata* (L.) Walp	Leguminosae
	10	*Artemisia*	Asteraceae
	2	*Brassica oleracea* L.	Rosaceae
	1	*Amorpha fruticosa* L.	Leguminosae
	8	*Potentilla supina* L. var. ternata Peterm.	Rosaceae
	2	*Lycopersicon esculentum* Mill.	Solanaceae
	18	*Humulus*	Moraceae
	5	Asteraceae	Asteraceae
*trn*H‐*psb*A	2	*Fraxinus chinensis* Roxb.	Oleaceae
	2	*Flueggea*	Euphorbiaceae
	7	Euphorbiaceae	Euphorbiaceae
	9	*Rumex*	Polygonaceae
	30	*Suaeda glauca* Bunge	Chenopodiaceae
	22	*Humulus*	Moraceae
	5	*Potentilla*	Rosaceae
	4	*Phaseolus vulgaris* L.	Leguminosae
	3	*Arachis hypogaea* L.	Leguminosae
	2	*Vitis*	Vitaceae
	1	*Descurainia sophia* (L.)Webb. ex Prantl	Brassicaceae
	1	*Vigna angularis* (Willd.) Ohwi et Ohashi	Leguminosae
	4	*Populus trichocarpa* Torr. & Gray	Salicaceae
	2	*Polygonum*	Polygonaceae
	2	*Agastache*	Labiatae
	16	*Ulmus*	Ulmaceae

**Table 2:** Percent success in extraction of plant DNA from gut contents and identification success of the resulting DNA sequences for the DNA barcodes *rbc*L, ITS, and *trn*H‐*psb*A

DNA barcodes	Amplicon size (bp)	Positive DNA detection (%)	Identification success per sequence (%)		
			Family	Genus	Species
*rbc*L	599	15.40 (37/240)	–	93.1	6.89
ITS	410	57.08 (137/240)	3.65	23.36	72.99
*trn*H‐*psb*A	430	42.10 (101/240)	6.25	53.57	40.18

Note

Numbers in parentheses represent the positive DNA detected number of samples/the total number of collected samples.

The combination of DNA data (using three DNA metabarcode markers) and distribution data for plants allowed us to identify 14 OTUs at the species level: *Gossypium hirsutum* L., *Suaeda glauca* Bunge, *Fraxinus chinensis* Roxb., *Potentilla supina* L. var. *ternata* Peterm., *Brassica oleracea* L., *Amorpha fruticosa* L., *Populus trichocarpa* Torr. & Gray, *Phaseolus vulgaris* L., *Arachis hypogaea* L., *Vigna angularis* (Willd.) Ohwi et Ohashi, *Descurainia sophia* (L.) Webb. ex Prantl, *Lycopersicon esculentum* Mill., *Vigna unguiculata* (L.) Walp, and *Amorpha fruticosa* L. (Table 1).

### 3.2 Feeding activity during different time periods

**Figure 2:**
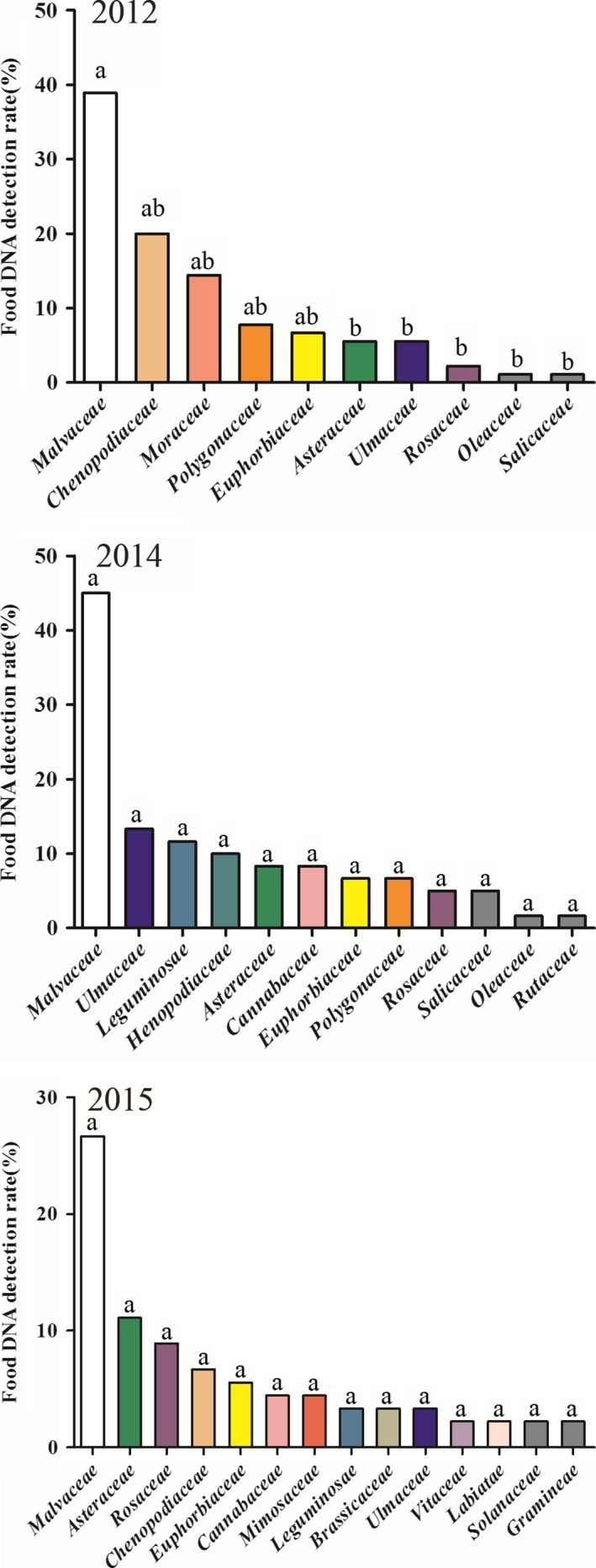
Detection rates of host plant DNA in *Apolygus lucorum* adults and the detected host plant genera in 2012, 2014, and 2015. Data from the sequence of DNA extracted from *A. lucorum* gut contents

Our analyses of the gut contents of adult individuals revealed that 35.9% of the oversea migratory *A. lucorum* were detected with the plant DNA from multiple hosts (*n* = 156), while the rest were found with that of only one host plant (Table 3). The detection rate of cotton DNA in *A. lucorum* was 35.8%, which was much higher than the detection rates of the other host plants (*F* = 6.42, *df* = 16,15, *p* = .0003) (Table 2, Figure 2).

**Table 3:** Percentage of *Apolygus lucorum* individuals feeding on different numbers of host plants

Year	Month	No. of samples	Percentage of *A. lucorum* feeding on different species of host plants (%)		
			1	2	3
2012	June	26	65.38 (17/26)	23.08 (6/26)	11.54 (3/26)
	July	18	66.67 (12/18)	11.10 (2/18)	22.20 (4/18)
	August	14	57.14 (8/14)	7.14 (1/14)	35.71 (5/14)
2014	June	20	80.00 (16/20)	15.00 (3/20)	5.00 (1/20)
	July	27	51.85 (14/27)	4.00 (1/27)	44.40 (12/27)
2015	June	13	61.53 (8/13)	30.77 (4/13)	7.70 (1/13)
	July	20	60.00 (12/20)	10.00 (2/20)	30.00 (6/20)
	August	18	77.80 (14/18)	5.60 (1/18)	16.70 (3/18)

Note

Numbers in parentheses represent the number of samples detected with different plant species/the total number of samples with positive plant DNA detection.

**Figure 3:**
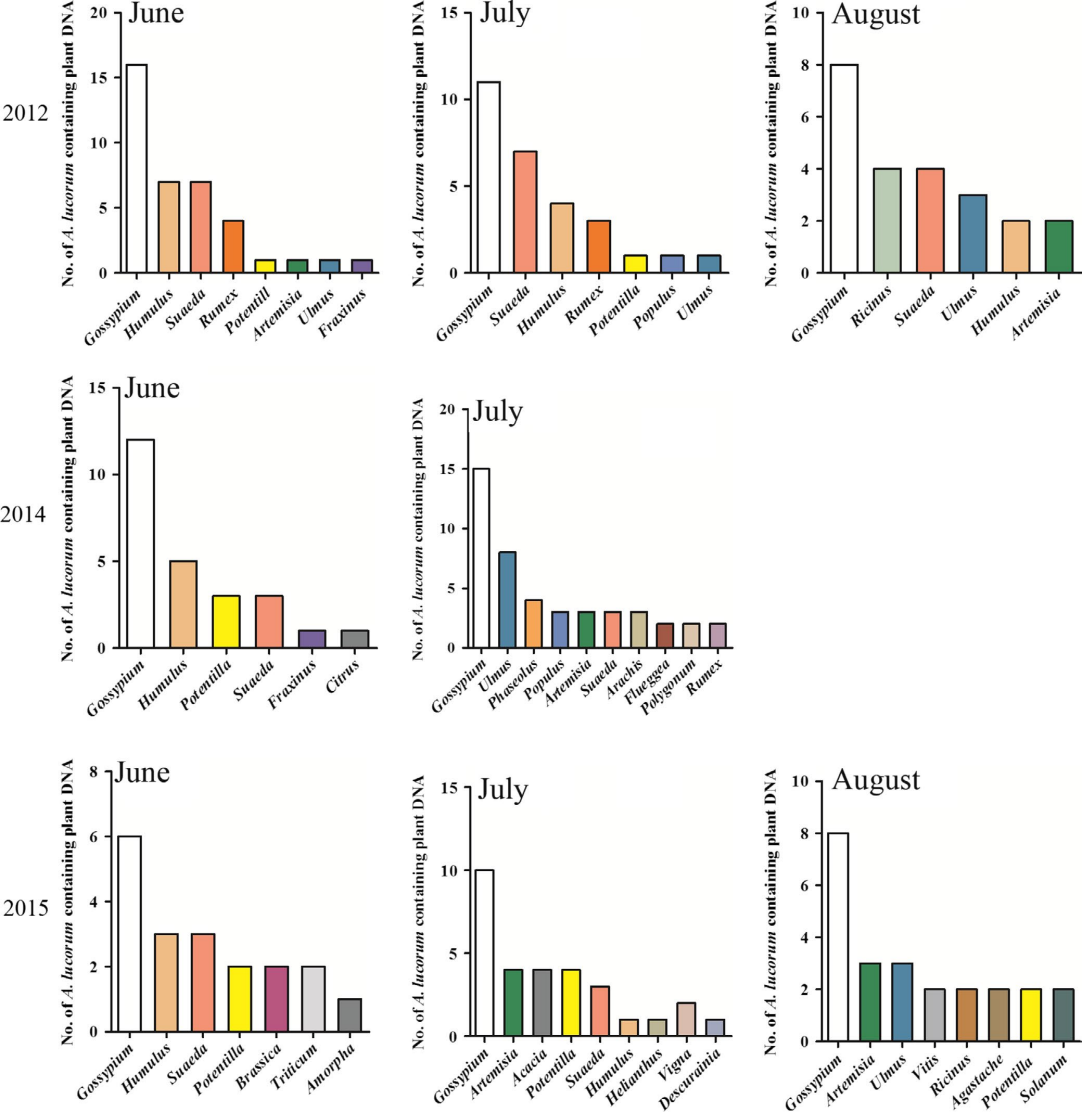
Number of *Apolygus lucorum* adults that contained plant DNA and the detected host plant genera in 2012, 2014, and 2015. Data from the DNA sequences extracted from *A. lucorum* gut contents

The host plants detected in *A. lucorum* were not significantly different among years (*F* = 2.24, *df* = 2,15, *p* = .1392). Four host plants were detected at a high frequency in adults in June 2012, 2014, and 2015: *G. hirsutum*, *Humulus* sp., *S. glauca*, and *Potentilla* sp. In July, more kinds of host plants were detected in adults (e.g., *P. vulgaris*, *A. hypogaea*, *P. trichocarpa*, *Artemisia* sp., and *Ulmus* sp.). In August 2012, 2014, and 2015, the most common host plants detected in adults were species of *Vitis*, *Ricinus*, and *Agastache*, as well as *L. esculentum* (Figure 3).

In 2012, a total of 8, 8, and 6 plant families were detected in the guts of *A. lucorum* adults in June, July, and August, respectively. The host plants detected in *A. lucorum* were significantly different among months (*F* = 5.85, *df* = 11,9, *p* = .0066). In June, *G. hirsutum* DNA was detected in 53.3% of individuals, while the percentages of bugs with DNA of *Humulus* sp. and *S. glauca* each equaled 23.3%. In July, *G. hirsutum* DNA was found in 36.7% of individuals, and *S. glauca* DNA was found in 23.3% of all adults. In August, the DNA of *G. hirsutum* (26.7%) and *Ricinus* sp. (13.3%) was the most prevalent detections (Figures 2 and 3).

In 2014, there were 6 and 8 families of host plant DNA detected in *A. lucorum* adults in June and July, respectively. The host plants detected in *A. lucorum* were not significantly different among months (*F* = 4.64, *df* = 14,1, *p* = .3505). In June, the DNA of *G. hirsutum* was detected in 40.0% of individuals, while that of *Ricinus* sp. was found in 16.7%. In July, *G. hirsutum* DNA was detected in 50.0% of all individuals, while DNA of *Ulmus* sp. was found in 26.7% of all bugs (Figures 2 and 3).

In 2015, a total of 7, 9, and 8 families of host plant DNA were detected in *A. lucorum* adults in June, July, and August, respectively. The host plants detected in *A. lucorum* were not significantly different among months (*F* = 2.53, *df* = 18,5, *p* = .1542). The detection rate of *G. hirsutum* DNA was the highest, totaling 20.0%, 33.3%, and 26.7% of all bugs with identified detections in these three months, respectively. In addition, the DNA of *Humulus* sp. and *S. glauca* was found in 10.0% of individuals in June, while that of Asteraceae was found in 13.3% (Figures 2 and 3).

## 4 DISCUSSION

In this study, we identified host plant families, genera, and species used by the oversea‐migrating adults of *A. lucorum* using DNA barcoding. We found that *A. lucorum* adults fed on a wide range of host plants, including at least 17 families. We also documented the simultaneous use of multiple host species by *A. lucorum* individuals.

The rapidly evolving sequences of the chloroplast genome region make them appropriate DNA barcodes for identifying plants (Valentini et al., 2009). The Consortium for the Barcode of Life (CBOL) working group has proposed the *rbc*L + *matK* combination as the best plant barcode because of its universality, sequence quality, and species discrimination (CBOL Plant Working Group, 2009). However, the success rate of plant DNA amplification in these mirid bugs was relatively low for the chloroplast *rbc*L intron (599 bp) in this study, probably due to degradation by extraoral digestion that reduced the number of larger DNA fragments remaining in the gut. Deagle, Eveson, and Jarman (2006) found that the number of template molecules of degraded DNA declined rapidly with increasing fragment size during the digestion period. Hereward and Walter (2012) suggested that the chloroplast *trn*L intron was not successfully amplified from target plant DNA in the green mirid bug *C. dilutus* because of degradation by extraoral digestion. *A. lucorum* resembles *C. dilutus* in feeding behavior, performing extraoral digestion and lacerating and macerating plant cells with a stylet‐probing movement and watery salivary discharge (Backus et al., 2007). In this study, we therefore selected the small regions ITS and *trn*H‐*psb*A, which are more suitable for PCR amplification of degraded DNA. The ITS and *trn*H‐*psb*A regions were amplified in 60.4% and 42.1% of *A. lucorum* samples, respectively. In addition, we successfully identified host plants to the genus (39.2%) and species (56.1%) levels. The successful extraction of plant DNA from gut contents and the adoption of multiple DNA markers (*rbc*L, ITS, and trnH‐psbA) made it possible to identify host plant associations to the genus (39.2%) and species (56.1%) levels. The success of García‐Robledo et al. (2013) in identifying host plants to the genus level was higher than that in other studies (Jurado‐Rivera et al., 2009; Pinzón‐Navarro, Barrios, Murria, Lyal, & Vogler, 2010) as they used more than one molecular marker. Our result is consistent with the findings for leaf‐rolling beetles (García‐Robledo et al., 2013), indicating that each of these three plant DNA barcode loci is not as universal as expected and that more than one locus should be used when reconstructing a network of herbivore–plant interactions.

Gut content amplicons can evidently be used to identify plant species within 12–48 hr postingestion (Fournier, Hagler, Daane, de León, & Groves, 2008; Gariepy, Kuhlmann, Gillott, & Erlandson, 2007; Hoogendoorn & Heimpel, 2001; Muilenburg, Goggin, Hebert, Jia, & Stephen, 2008). For *A. lucorum*, we conducted plant feeding trials of mirids that were starved for 48 hr to confirm that no plant tissues remained within their guts and found that plant DNA detection gradually declined with increased digestion time immediately after feeding and that the maximum digestion time (the point at which detection was no longer possible) of four tested plants (cotton, *Humulus scandens*, *Medicago sativa*, and *Vigna radiata*) was >16 hr postfeeding (Wang, Bao, Yang, Xu, & Yang, 2017; Wang et al., 2018). A previous study found that *A. lucorum* adults were most active from 16:00 to 24:00 in crop fields (Geng, Lu, & Yang, 2012). Therefore, we speculated that the time of *A. lucorum* adult flight from host plants was at dusk. As we collected *A. lucorum* adults from the light traps at 6:00 every morning, DNA analysis took place approximately 6–12 hr after the last time of plant feeding of *A. lucorum* before it began its migration over the sea. The number of template molecules of the degraded DNA declined rapidly with increasing fragment size during the digestion period (Deagle et al., 2006; Hereward & Walter, 2012; Wallinger et al., 2013; Wang, 2017; Wang et al., 2018). Hence, we targeted short DNA fragments of multiple‐copy genes to increase the probability of successful DNA detection (Traugott et al., 2013). Plant DNA recovery rates from the gut contents of *A. lucorum* collected on Beihuang Island ranged between 42.1% and 60.4%, which was higher than in some previous insect–plant trophic interaction studies using DNA sequencing. For example, García‐Robledo et al. (2013) found plant DNA recovery rates from the gut contents of leaf‐rolling beetles ranging between 45.9% and 48.7%. Navarro et al. (2010), in contract, found a DNA recovery rate from weevils of just 35.6%. However, the values were much larger for weevils and leaf beetles (66% of leaf beetles and 67% of weevils) collected directly during their foraging and preserved immediately for DNA analyses (Kajtoch, Kubisz, Heise, Mazur, & Babik, 2015). These samples were immediately preserved in the field in ethanol to minimize DNA degradation. Our study demonstrates that it is possible to determine the host use and ultimately dietary breadth of migratory insects from herbivore tissue by DNA‐based plant identification.

In this study, a significant proportion of *A. lucorum* individuals were found to have fed on multiple host plants. Fragments of the length that we amplified from the mirid gut contents can evidently be detected only within 48 hr postingestion (Fournier et al., 2008; Gariepy et al., 2007; Hoogendoorn & Heimpel, 2001; Muilenburg et al., 2008). Therefore, individual mirid adults frequently move between hosts. Similarly, *A. lucorum* individuals moved frequently between cotton and mungbean fields when these crops were planted nearby (Wang, 2017). Moreover, *Creontiades dilutus* (Hemiptera: Miridae) often feeds on several host plant species other than the one it has been collected from, based on molecular gut content analyses (Hereward, 2012; Hereward & Walter, 2012), indicating potential movement and the utilization of multiple host plants by this mirid bug. *Nezara viridula* (Hemiptera: Pentatomidae) showed similar feeding habits, moving from one plant species to another during the feeding process (Todd, 1989), while host switching enhanced its survival and reproduction (Velasco & Walter, 1993). For *A. lucorum*, Pan, Liu, and Lu (2018) found that the combination of feeding nymphs on maize and adults on green bean resulted in the fastest population growth rate in the laboratory, indicating that host food switching between stages was beneficial. This potential benefit warrants further investigation under natural conditions to determine whether the ecological significance of *A. lucorum* movement resembles that of *N. viridula*.

As a polyphagous species, *A. lucorum* has been recorded on at least 288 different host species in 54 different families (Jiang et al., 2015). Based on our analyses of the gut contents of individual adults, *A. lucorum* fed on hosts from at least 17 plant families. Among these hosts, the species *F. chinensis*, *Citrus*, and *P. trichocarpa* had not been recorded in previous studies. *F. chinensis* and *P. trichocarpa* are deciduous trees, and both of them and some *Citrus* species are widely distributed in northern China. This finding indicates a potentially wider host range of *A. lucorum* than previously thought. In Beihuang Island, there is no plant species which have detected from *A. lucorum*'s gut content in this study. It showed strong evidence of oversea migration of *A. lucorum* (Fu et al., 2014) and then provided important information on host plant use of *A. lucorum* population migrated from the land of northern China.

In northern China, *A. lucorum* usually undergoes five generations each year, emerging from overwintering host plants (some weeds and fruit trees) in mid‐April, developing to the adult stage on early‐season host plants close to the overwintering sites, and then spreading to cotton fields by mid‐June. The third and fourth generations of nymphs are mainly damaging to cotton fields. With the deterioration of food conditions in cotton fields, most fourth‐generation adults migrate to other plants in September (Lu & Wu, 2008). According to our molecular analyses of the gut contents of individual adult bugs, cotton is the dominant host plant of adults, followed by various weeds from June to August. The weed species were mainly *S. glauca* and *Humulus* sp. from June to July, while more kinds of weeds (e.g., species of *Ricinus* and *Agastache*) were detected in adults in August. In addition, *A. lucorum* also migrated onto Leguminosae (e.g., *P. vulgaris*, *V. angularis*, *V. unguiculata*, and *A. hypogaea*) and Asteraceae (e.g., species of *Artemisia* and *Helianthus*) when these plants were at the flowering stage and fed on them during July and August. Our results also suggest that a small number of mirid bugs feed on woody plants. According to previous field surveys, *A. lucorum* adults prefer some plant species when they are in bloom, such as *Vigna radiata*, *G. hirsutum*, *Helianthus annuus*, and *Chrysanthemum coronarium* in early July; by late July, adults disperse to other flowering hosts (e.g., *Ricinus communis*, *Impatiens balsamina*, *Humulus scandens*, *Ocimum basilicum*, and *Agastache rugosua* (Lu, Wu, Wyckhuys, & Guo, 2010; Pan et al., 2013)). Our results are consistent with previous findings.

In summary, we identified the diets of migratory mirid bugs by multiple DNA barcode loci at the plant family, genus, and species levels. Our findings suggest that *A. lucorum* individuals feed on multiple host plants. This is a significant step in studying the feeding ecology of *A. lucorum* under natural conditions and developing landscape‐level pest management strategies for this mirid bug.

## CONFLICT OF INTEREST

None declared.

## AUTHOR CONTRIBUTIONS

YHL and YZY conceived the idea and designed the methodology; XWF collected the samples; QW, WFB, and QZ performed the laboratory work; QW analyzed the data; and QW and YHL wrote the manuscript.

## Data Availability

Sequence files have been deposited in the Dryad data repository (https://doi.org/10.5061/dryad.9cp7219).
