# Supplementary material for: Host plant use of a polyphagous mirid, Apolygus lucorum: Molecular evidence from migratory individuals
Source: Ecol Evol. 2019 Sep 21;9(19):11518–28. doi: 10.1002/ece3.5660 (PMC6802376; doi:10.1002/ece3.5660)
Supplement: Supplementary file 1 [file ECE3-9-11518-s001.doc]

**Supporting Information**

**Table S1. Primers used in this study to obtain DNA sequences from insect gut contents**

| **Primer** | **Sequence** | **Reference** |
| --- | --- | --- |
| ITS1-F-rc* | GACTCTCGGCAACGGATATC | (Baamrane et al., 2012) |
| ITS4 | TCCTCCGCTTATTGATATGC | (White, Bruns, Lee, & Taylor, 1990) |
| *rbcL*_F | ATGTCACCACAAACAGAGACTAAAGC | (Soltis, Soltis, & Smiley, 1992) |
| *rbcL*_Rev | GTAAAATCAAGTCCACCRCG | (Kress, Erickson, Jones, Swenson, Perez, Sanjur, & Bermingham, 2009) |
| *psb*A | GTTATGCATGAACGTAATGCTC | (Sang, Crawford, & Stuessy, 1997) |
| *trn*H | CGCGCATGGTGGATTCACAATCC | (Tate & Simpson, 2003) |

**References**

Baamrane, M. A. A., Shehzad, W., Ouhammou, A., Abbad, A., Naimi, M., Coissac, E., . . . Znari, M. (2012). Assessment of the food habits of the Moroccan dorcas gazelle in M'Sabih Talaa, west central Morocco, using the trnL approach. *PLoS ONE, 7*(4), e35643.

Sang, T., Crawford, D., & Stuessy, T. (1997). Chloroplast DNA phylogeny, reticulate evolution, and biogeography of *Paeonia* (Paeoniaceae). *American Journal of Botany, 84*(8), 1120-1136.

Soltis, P. S., Soltis, D. E., & Smiley, C. J. (1992). An rbcL sequence from a Miocene Taxodium (bald cypress). *Proceedings of the National Academy of Sciences of the United States of America, 89*(1), 449-451.

Tate, J. A., & Simpson, B. B. (2003). Paraphyly of Tarasa (Malvaceae) and diverse origins of the polyploid species. *Systematic Botany, 28*(4), 723-737.

White, T. J., Bruns, T., Lee, S., & Taylor, J. (1990). Amplification and direct sequencing of fungal ribosomal RNA genes for phylogenetics. In M. A. Innis, D. H. Gelfand, J. J. Sninsky, & T. J. White (Eds.), *PCR Protocols* (pp. 315-322). San Diego: Academic Press.

Kress, W. J., Erickson, D. L., Jones, F. A., Swenson, N. G., Perez, R., Sanjur, O., & Bermingham, E. (2009). Plant DNA barcodes and a community phylogeny of a tropical forest dynamics plot in Panama. *Proceedings of the National Academy of Sciences of the United States of America, 106*(44), 18621-18626.
